# Nutrition by Design: Boosting Selenium Content and Fresh Matter Yields of Salad Greens With Preharvest Light Intensity and Selenium Applications

**DOI:** 10.3389/fnut.2021.787085

**Published:** 2022-01-05

**Authors:** Xudong Zhu, Tianbao Yang, Charles A. Sanchez, Jeffrey M. Hamilton, Jorge M. Fonseca

**Affiliations:** ^1^Food Quality Laboratory, Beltsville Agricultural Research Center, Agricultural Research Service, United States Department of Agriculture, Beltsville, MD, United States; ^2^Department of Environmental Sciences, Maricopa Agricultural Center, University of Arizona, Maricopa, AZ, United States; ^3^Arid Lands Resource Sciences, University of Arizona, Tucson, AZ, United States

**Keywords:** arugula, leafy greens, lettuce, rocket, solar radiation

## Abstract

Selenium (Se) is an essential mineral in multiple human metabolic pathways with immune modulatory effects on viral diseases including the severe acute respiratory syndrome coronavirus 2 (SARS-CoV-2) and HIV. Plant-based foods contain Se metabolites with unique functionalities for the human metabolism. In order to assess the value of common salad greens as Se source, we conducted a survey of lettuce commercially grown in 15 locations across the USA and Canada and found a tendency for Se to accumulate higher (up to 10 times) in lettuce grown along the Colorado river basin region, where the highest amount of annual solar radiation of the country is recorded. In the same area, we evaluated the effect of sunlight reduction on the Se content of two species of arugula [*Eruca sativa* (*E. sativa*) cv. “Astro” and *Diplotaxis tenuifolia* (*D. tenuifolia*) cv. “Sylvetta”]. A 90% light reduction during the 7 days before harvest resulted in over one-third Se decline in *D. tenuifolia*. The effect of light intensity on yield and Se uptake of arugula microgreens was also examined under indoor controlled conditions. This included high intensity (HI) (160 μ mol^−2^ s^−1^ for 12 h/12 h light/dark); low intensity (LI) (70 μ mol m^−2^ s^−1^ for 12 h/12 h light/dark); and HI-UVA (12 h light of 160 μ mol m^−2^ s^−1^, 2 h UVA of 40 μ mol m^−2^ s^−1^, and 10 h dark) treatments in a factorial design with 0, 1, 5, and 10 ppm Se in the growing medium. HI and HI-UVA produced *D. tenuifolia* plants with 25–100% higher Se content than LI, particularly with the two higher Se doses. The addition of Se produced a marked increase in fresh matter (>35% in *E. sativa* and >45% in *D. tenuifolia*). This study (i) identifies evidence to suggest the revision of food composition databases to account for large Se variability, (ii) demonstrates the potential of introducing preharvest Se to optimize microgreen yields, and (iii) provides the controlled environment industry with key information to deliver salad greens with targeted Se contents.

## Introduction

The micronutrient selenium (Se) has attracted much attention of medical researchers during the last several decades. Over 30 mammalian selenoproteins (1, 2) have been identified, many of which provide biochemical disease resistance for mammalian health (3–5). It has been suggested that the intake of Se reduces the risk of chronic health problems and skeletal deformities (6, 7) and metastasis of diverse cancer tumors (8, 9) including those associated to esophageal and gastric cancer (10). For example, in England and Japan, contemporary dietary changes to foods with lower Se content were correlated with a higher incidence of cancer and chronic diseases (9, 11).

Se deficiency has also been shown to increase RNA replication of various viruses (12), which has elicited attention of the scientific community due to the emergence of coronavirus disease 2019 (COVID-19) pandemic caused by the severe acute respiratory syndrome coronavirus 2 (SARS-CoV-2). The deficiency of the selenoprotein P has been found remarkably efficient to predict risk of death for COVID-19 (13, 14). A recent report suggested the biofortification of plants to produce a broad impact on COVID-19, arguing that plant-derived Se compounds can be a significant supplementary treatment for prevention and disease control and can be an effective way to reduce massive viral load and subsequent mutation of the virus (15).

Despite the abundance of peer-reviewed literature revealing the beneficial role of dietary Se in humans and the relationship between a diet deficient in Se with many health problems (16–18), there is increasing prevalence of Se deficiency in many areas of the world (9, 18). On the other hand, in North America, the common availability of Se supplements poses the risk of overdosage (19, 20).

Both the inadequate dietary Se and excessive Se consumption from supplements pose human health risks (21). However, targeting an optimal recommended dietary allowance (RDA) for Se is a challenge because Se is one of the minerals with the narrowest dose range between toxicity and deficiency. A minimum Se range between 15 (infants) to 70 μg (lactating women) is considered essential for daily intake (22). Higher Se intake levels than the RDA could be necessary for Se to be effective for specific health conditions (21) including a suggested daily 2 mg dose for specific cancer prevention (23). Considering that Se is highly toxic when its daily concentration reaches 4 mg (24, 25), Se intake via food consumption appears the safest approach for most people.

Significant content of Se is found in food with low water fraction and high protein fraction such as Brazil nuts, walnut, peanut, garlic, onion, chicken, meat, and seafood (26, 27). Plant-based foods contain a variety of Se forms such as inorganic selenite and selenite, seleno amino acids, and monomethylated Se-methylselenocysteine (SeMScys) and γ-glutamyl-Se-methylselenocysteine (GGSeMSCys). In organic form, Se can induce oxidative stress and produce malformed selenoproteins, in addition to affecting protein normal function when present in excess. However, SeMSCys cannot be integrated into proteins and is efficiently converted in the body to non-toxic, chemopreventive methylselenol. SeMSCys was found to be the major selenocompound in selenium-enriched plants such as those in the genus *Brassica* (28). This suggests that these vegetables can be a qualitative and safe source of Se. The contribution of vegetables can also be significant in quantitative terms. In the Sonoran desert region of northern Mexico, vegetables contribute as much as 28–32% of the daily overall Se intake (29).

Preharvest conditions appear to influence the concentration of Se in vegetables. While it may be expected that availability of Se in the soil has an influence on the final Se content of a harvested tissue, little is known about other preharvest factors. In New Zealand (30) and Finland (31, 32), Se is added to fertilizers, which has been linked to an increase of Se in the diet. However, fertilization needs to be applied at an adequate dosage and timed appropriately, since levels higher than 29 ppm in the soil inhibit the germination of vegetable seeds (33).

Therefore, our objective was to obtain a broad view of the Se content in economically important salad greens and identify any preharvest factors that could influence the final Se content. Subsequently, we aimed to determine whether light intensity has any potential effect on the accumulation of Se in leaves and petioles, using arugula as a model. In parallel to the latter, we investigated the potential effect of Se and light (or any potential interaction of them) on fresh matter yields.

## Materials and Methods

### Commercially Grown Lettuce Sampling

Since lettuce (*Lactuca sativa*) is one of the most consumed vegetables in the USA (34), we selected this crop for a broad preliminary screening for Se in leafy vegetables. Lettuce plants were sourced from 15 different locations across the USA and Canada. The lettuce types included red leaf, green leaf, Boston, romaine, and iceberg. The locations and type of lettuce were selected for their prominence in the local/national market. Thus, production locations or types of lettuce associated with small volumes in the market were not considered in this study. Supplementary Table 1 shows the number of samples and type of lettuce from each location considered for this study. Further information with respect to Se content in soil in the countries covered by this study is given in Supplementary Table 2.

The lettuce was harvested from commercial fields, prepared as commercial products (e.g., wrapper leaves were removed from heads of iceberg and romaine lettuce plants), and immediately placed in Styrofoam coolers containing dry ice. A layer of corrugated board was placed between the lettuce and the dry ice.

The samples were shipped in coolers and delivered via Express Courier Service on the next day to the Soil and Water Laboratory of the University of Arizona Yuma Agricultural Center. With the exception of lettuce sampled from the Coachella Valley (California), which was carried to the research station on the day after harvest, the samples collected from the Colorado river regions were brought to the laboratory on the same day. Upon arrival at the laboratory, the samples were immediately placed in a freezer at −20°C until the samples were freeze dried.

### Arugula Cultivation and Experimentation in Open Fields

To further study in this phase, we selected the two best known arugulas species for their diverse economic value in different areas of the world, with the understanding that both are cruciferous crops that can easily accumulate Se. *Eruca sativa* (*E. sativa*) (or salad rocket) is an annual crop that has become popular as a ready-to eat vegetable salad in the USA. The perennial “wild rocket” [*Diplotaxis tenuifolia* (*D. tenuifolia*)], while common in the Mediterranean region, is not so common in the USA. Our preliminary field trials showed that *D. tenuifolia* had higher antioxidant values than the commercially available *E. sativa*, another reason for considering both species. The morphology of the leaves of both type plants is shown in Supplementary Figure 1.

Seeds of these arugulas (*E. sativa* cv. Astro and *D. tenuifolia* cv. Sylvetta) were sourced from Johnny's Selected Seeds, Winslow, Maine, USA and grown during Fall (October to November) and Spring (March to April) seasons, at the University of Arizona Yuma Agricultural Center (Yuma, Arizona, USA) in deep coarse to fine-textured, alluvial loamy-clay hyperthermic arid soils. The average soil temperatures were 9–12°C during Fall (preliminary trial) and 19–30°C during Spring. Other information about weather in the area is given in Supplementary Table 3.

The crops were subjected to agronomic practices as applied in commercial settings in the area. Fertilization included 31 L-Ha^−1^ of 0N-52P-0K in preplant stage, followed by 3 side-dress injections of UAN-32 (32N-0P-0K) at 38 L-Ha^−1^. Irrigation water schedule was as follows: initial water for seed germination was provided through overhead sprinkling and once the seedlings were established, water was brought to the field through furrow irrigation. All the treatments received the same amount of water during the season (~30 cm) and were harvested on the same day. Harvests were done during early morning (7:00–10:00 a.m.), and took place 38 (*E. sativa*) and 50 days (*D. tenuifolia*) after planting when plants were considered mature and in preflowering stage. Growth period was equivalent to 1,100–1,600 accumulated degree days, using 4.4°C as the base temperature.

The reduction of sunlight received by the plants was accomplished by a bottom-opened box with nylon shade cloth in 5 sides that reduced the light intensity by ~60 and 90%. The boxes were rectangular wood-frame structures (0.75 m width × 0.5 m height × 1.5 m length). Supplementary Figure 2 is a photograph of the boxes in the field. Information about the boxes and the microenvironment they produced is given in Supplementary Table 4. The reduction of light was implemented 7 days before harvest. The experimental design was constructed with a complete randomized distribution of treatments along plots. There were 13 beds (182 m long, 101.6 cm wide) that in an alternating sequence had each of the two crops and buffer beds at each extreme of the plots and in between the planted beds.

### Arugula Microgreens Experimentation Under Controlled Growing Conditions

In the next phase of this study, our focus was to obtain results in indoor controlled conditions. Seeds of arugula *E. sativa* cv. Astro and *D. tenuifolia* cv. Sylvetta were treated with a solution of 20% chlorine bleach for 10 min and then were spread evenly over a hydroponic “Sure to Grow Pads” (0.508 m × 0.254 m, Growers Supply, USA). Seeds were watered with sodium selenate solution of 0 (control), 1, 5, and 10 ppm, respectively. The trays were kept in a growth chamber at 25°C under dark for 4 days. From the 5th day, seedlings were exposed to different light settings: high intensity (HI) (160 μ mol m^−2^ s^−1^ for 12 h/12 h light/dark); low intensity (LI) (70 μ mol m^−2^ s^−1^ for 12 h/12 h light/dark); and HI-UVA (12 h light of 160 μ mol m^−2^ s^−1^ plus 2 h UVA, 385 nm, of 40 μ mol m^−2^ s^−1^ plus 10 h dark). The *E. sativa* microgreens were harvested on the 14th day and *D. tenuifolia* plants were harvested on the 15th day by cutting the plants about 1 cm above the pad surface and weighed to obtain their fresh weight.

### Measurements of Selenium Content in Plants

The plants grown in open fields were diced and mixed thoroughly. A subsample was reserved in the freezer. Main samples were freeze dried. After drying, the samples were kept in the freezer until ready for analyses. In preparation for analyses, the samples were ground and stored in vials for digestion according to previously reported protocols (35). The ground plant materials were digested using concentrated nitrite hydrate (H_2_NO_3_) and hydrogen peroxide (H_2_O_2_) block digestion.

The digests were analyzed for total Se content by inductively coupled plasma mass spectrometry (ICP-MS). For this procedure, the samples were brought to dryness and sent to the Arizona Laboratory for Emerging Contaminants (ALEC) (Tucson, Arizona, USA) for the mineral analysis. Two genuine standards of Se (Supelco, 20 and 100 μg) were also included for comparison with each set of samples analyzed. Samples of dried asparagus and arugula, spiked with a known concentration of Se, were also part of the samples, to determine accuracy.

The microgreens plants (three replicates) grown in controlled conditions were harvested on the indicated days and fresh samples were analyzed for Se content by the Eurofins Microbiology Laboratories (Lancaster, Pennsylvania, USA). The samples were digested with microwave under pressure in concentrated nitric acid. The digested samples were then subjected to ICP-MS quantification. The Se signal in each sample was compared to a set of the National Institute of Standards and Technology (NIST) traceable standards to determine the concentration.

### Statistical Analysis

Se content data for triplicate samples of lettuce and arugula were subjected to the ANOVA at *p* ≤ 0.05 to determine statistical significance. Mean comparisons were conducted using the Fisher's protected least significant difference (LSD) method at *p* ≤ 0.05 (SAS Institute, Cary, North Carolina, USA).

The experiments evaluating the effect of shading (sunlight reduction) on Se content were arranged in a completely randomized design and each treatment consisted of 3 replicates. When appropriate, the data were subjected to the Student's *t*-test or the (ANOVA) at *p* ≤ 0.05 to determine statistical significance; if significant differences were observed, mean separation was carried out by the Duncan's multiple range test at *p* ≤ 0.05.

For the analysis of the light effect under controlled conditions, the fresh weight and Se data were analyzed by the two-way ANOVA and the Duncan's multiple range test at a significance level of *p* ≤ 0.01.

## Results

### Se Content in Lettuce Across North America

**Figure 1:**
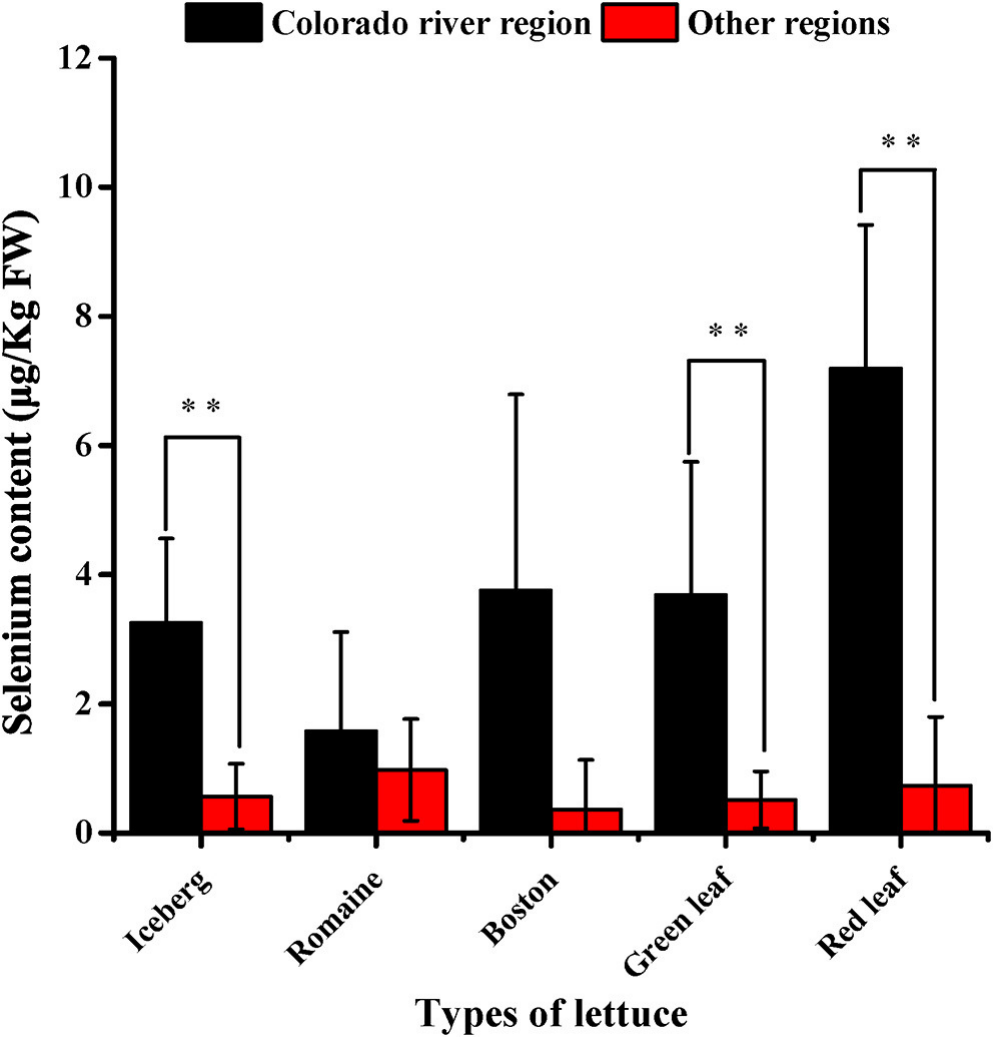
Selenium content in North America lettuce. Error bars are SD of the mean. Number of samples were 108 from the Colorado River Basin and 26 from other regions including those known as US Pacific (e.g., California), US Mountain (e.g., Colorado), US North Central (e.g., Ohio, Michigan), US Mid-Atlantic/Northeast (e.g., New Jersey, New York), and Southeastern Canada (e.g., Montreal). For more details, see Supplementary Table 1. Two star indicates significant differences between treatment means at *p* < 0.01.

In the survey of commercially grown lettuce, our results showed that the plants grown along the Colorado river basin have significantly higher Se content than lettuce plants grown elsewhere (Figure 1). The exceptions to this were observed with romaine and Boston lettuce, for which data showed high variability. The other types of lettuce grown on the Colorado river basin had over 4 (iceberg), 5 (green leaf), and 10 (red leaf) fold increase in Se content. These results suggested the critical effect of light intensity given that this region continuously shows the highest recorded solar radiance (36), whereas temperature and soil Se content (Supplementary Table 2) are variable across all the locations.

The results obtained from plants grown in the Colorado river basin region also revealed differences among types of lettuce. Red leaf lettuce had significantly higher Se content than iceberg and romaine lettuce. In the other locations/regions, we could not identify any difference among the lettuce types because for some types, the sample was too small or not all the types were represented in those locations.

### Se Contents in Arugulas Under Open Field Conditions

**Figure 2:**
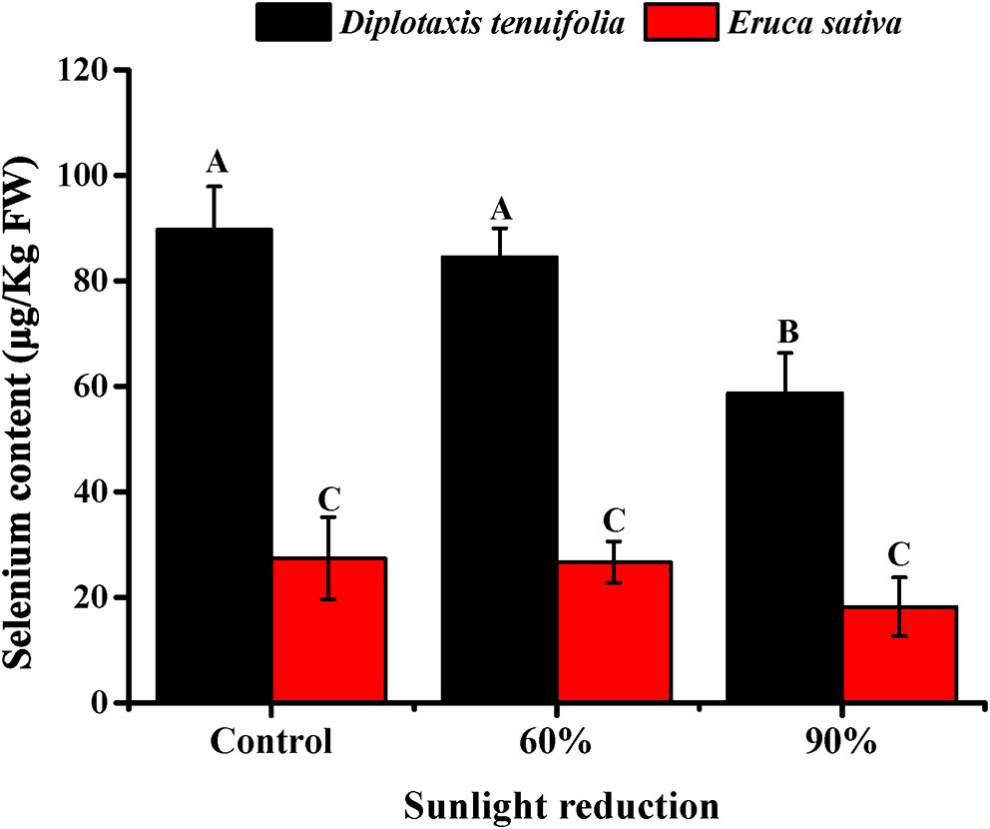
Effect of sunlight reduction during last 7 days before harvest on leaf Se content in *Diplotaxis tenuifolia* cv. Sylvetta and *Eruca sativa* cv. Astro grown during fall season (October to November) in Yuma, Arizona, USA. Data presented are the means of three replicates. Error bars indicate SD of the means. Different letters (A–C) indicate significant differences between treatment means at *p* < 0.01.

To investigate the effect of light intensity, we selected two arugulas (“salad rocket” and “wild rocket”) for further investigation, given the capability of the two *Brassica* species to accumulate Se. The results obtained for arugula plants showed that *D. tenuifolia* tend to accumulate much higher Se than *E. sativa* under the typical desert conditions in Arizona (Figure 2). The content of Se in *D. tenuifolia* ranged from 58 to 90 μg/kg, while the content of Se in *E. sativa* ranged from 21 to 27 μg/kg. The reduction of light by 60% did not cause any significant change in Se content in either species of arugula. However, the reduction of light by 90% produced a notable decline in Se content, particularly in *D. tenuifolia*. In this case, the plants subjected to the 7-day light reduction contained over one-third lower Se content than that observed in the control plants. A similar trend was observed earlier in the preliminary trial (data not shown).

### Effects of Light Intensity and Se Application on the Yield of Arugula Microgreens in Indoor Controlled Conditions

**Figure 3:**
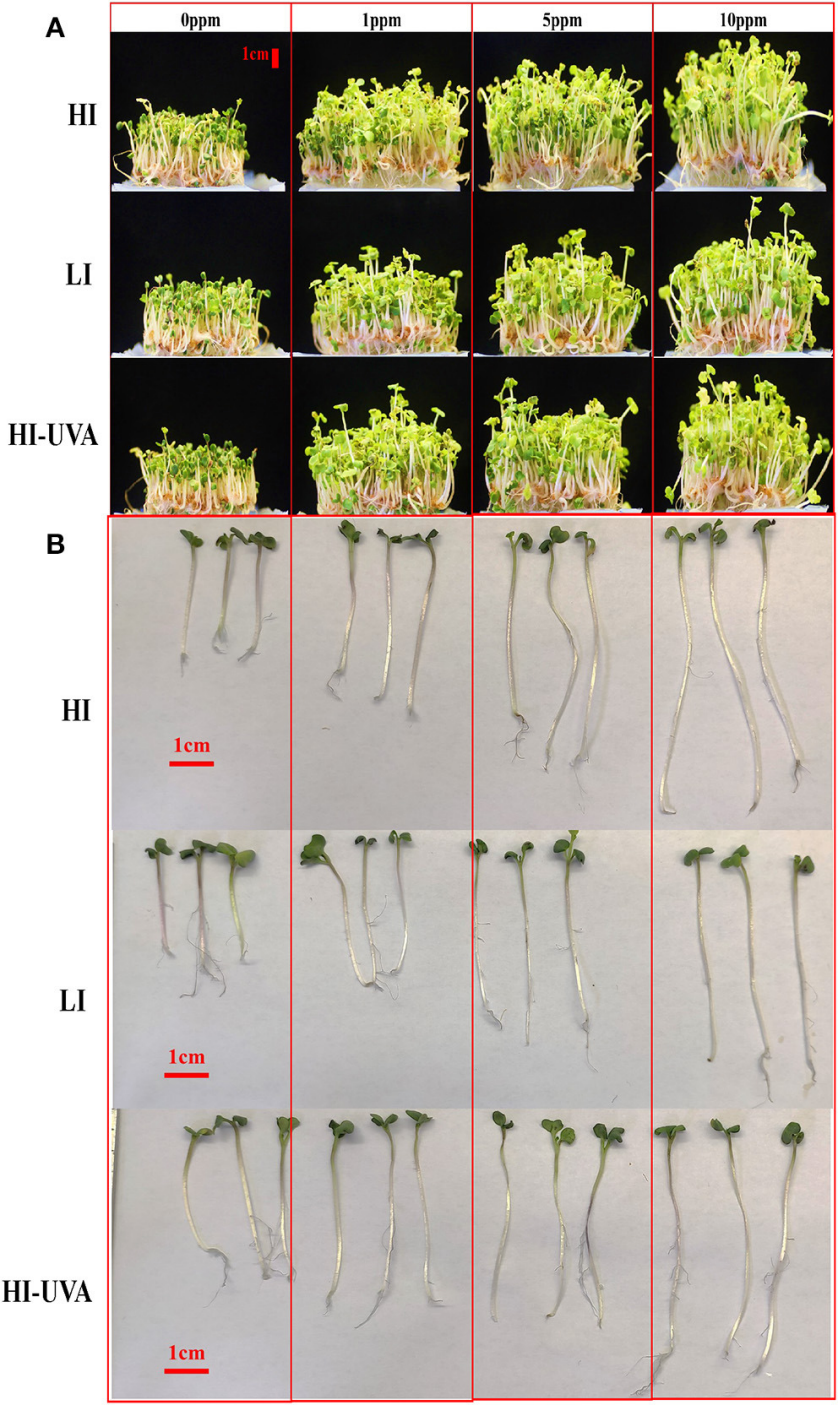
Effects of light and selenium application on the growth of *Eruca sativa* cv. Astro in a controlled environment. **(A)** Growth status of seedlings. **(B)** Comparison of hypocotyl length. Plants were grown in a growth chamber at 25°C with different light settings. HI, high-light intensity, 160 μ mol m^−2^ s^−1^ for 12 h/12 h light/dark; LI, low-light intensity, 70 μ mol m^−2^ s^−1^ for 12 h/12 h light/dark; HI-UVA, high-light intensity plus UVA (385 nm), 160 μ mol m^−2^ s^−1^ for 12 h, 40 μ mol m^−2^ s^−1^ for 2 and 10 h dark. Photos were taken on harvest day (day 14).

**Figure 4:**
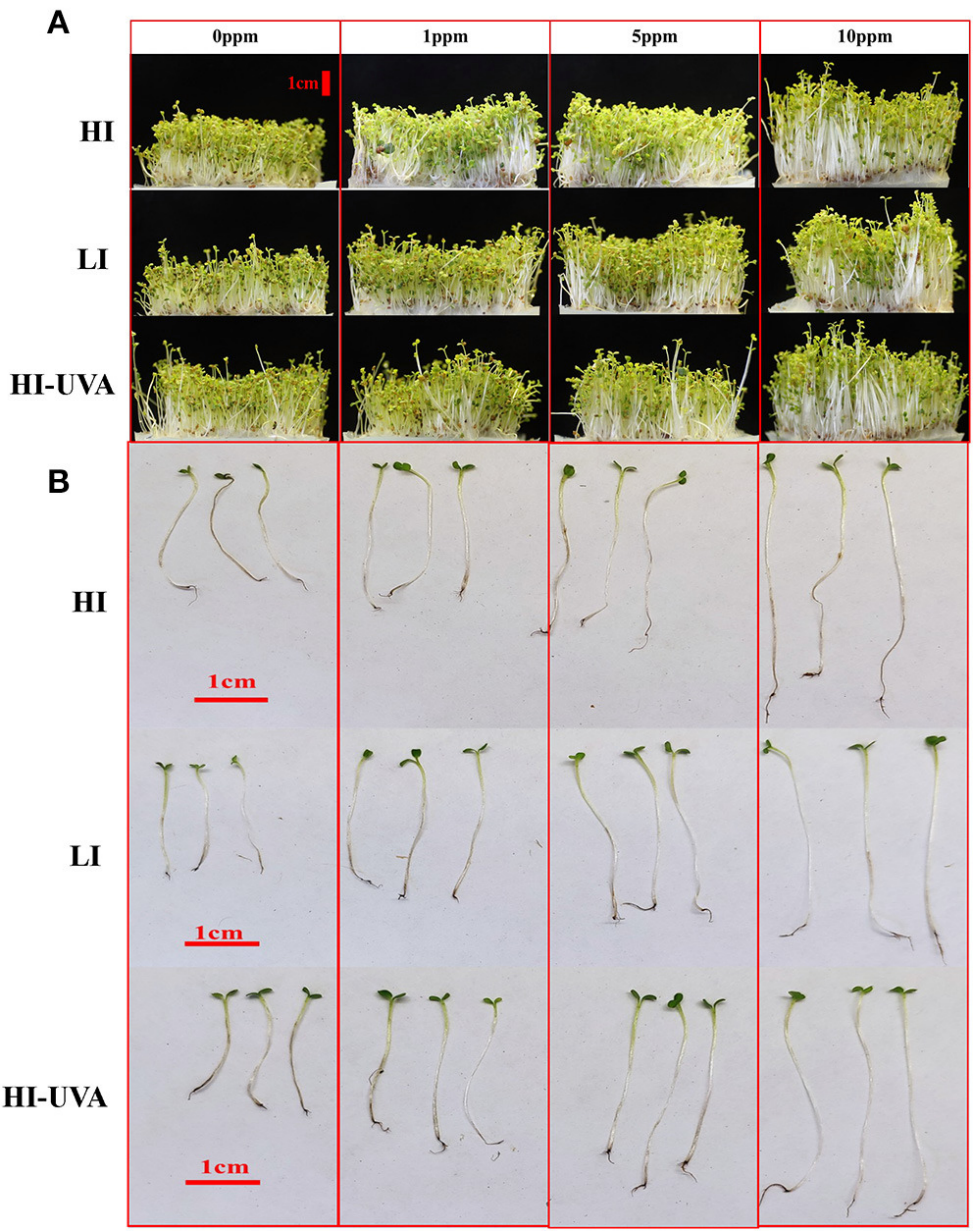
Effects of light and selenium application on the growth of *Diplotaxis tenuifolia* cv. Sylvetta in a controlled environment. **(A)** Growth status of seedlings. **(B)** Comparison of hypocotyl length. Plants were grown in a growth chamber at 25°C with different light settings. HI, high-light intensity, 160 μ mol m^−2^ s^−1^ for 12 h/12 h light/dark; LI, low-light intensity, 70 μ mol m^−2^ s^−1^ for 12 h/12 h light/dark; HI-UVA, high-light intensity plus UVA (385 nm), 160 μ mol m^−2^ s^−1^ for 12 h, 40 μ mol m^−2^ s^−1^ for 2 and 10 h dark. Photos were taken on harvest day (day 15).

**Figure 5:**
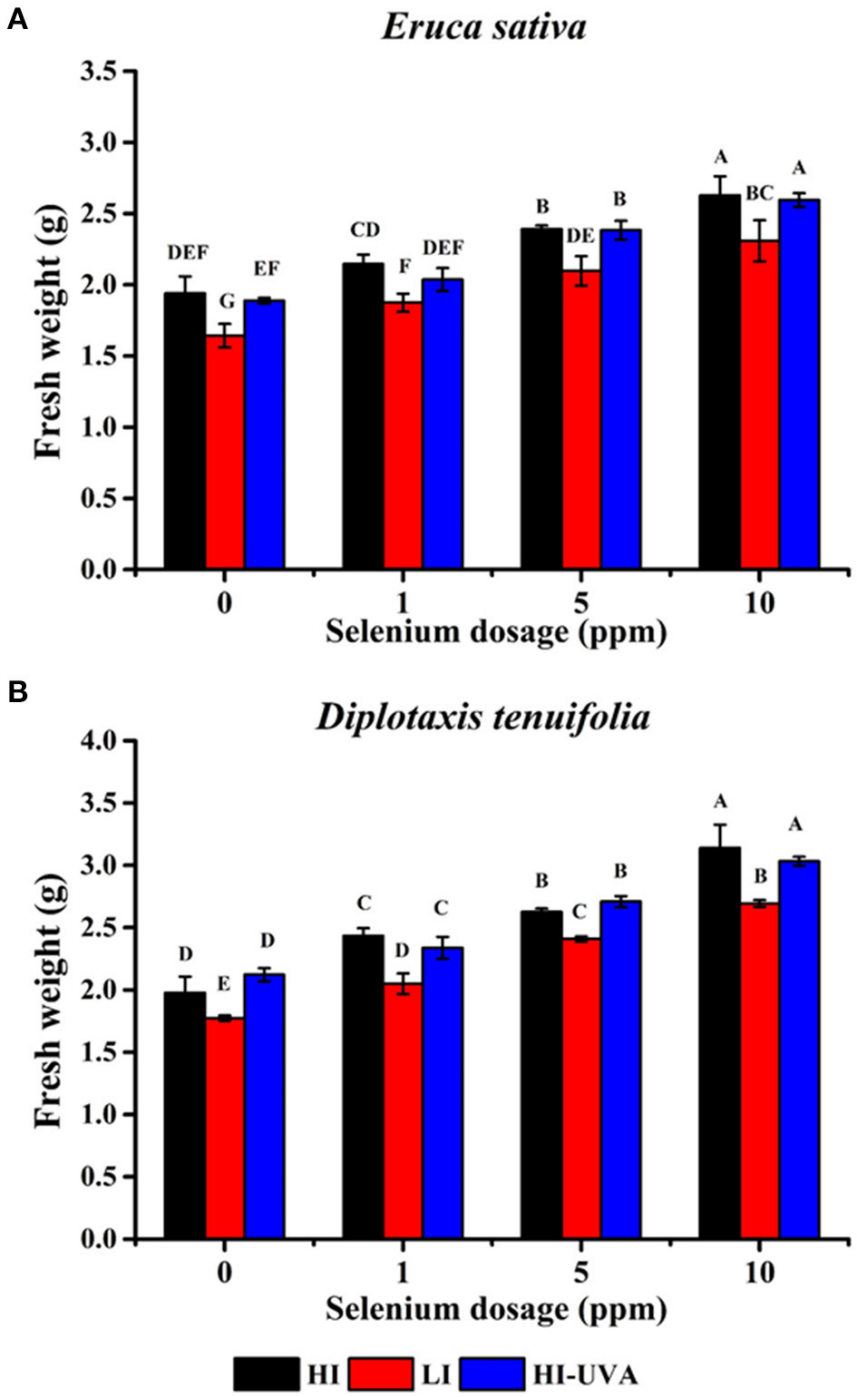
Effects of light and selenium application on microgreens fresh weight of *Eruca sativa cv*. Astro **(A)** and *Diplotaxis tenuifolia* cv. Sylvetta **(B)** in a controlled environment. Plants were grown in a growth chamber at 25°C with different light settings. HI, high-light intensity, 160 μ mol m^−2^ s^−1^ for 12 h/12 h light/dark; LI, low-light intensity, 70 μ mol m^−2^ s^−1^ for 12 h/12 h light/dark; HI-UVA, high-light intensity plus UVA (385 nm), 160 μ mol m^−2^ s^−1^ for 12 h, 40 μ mol m^−2^ s^−1^ for 2 and 10 h dark. Data presented are the means of three replications of fresh weight (g/g seeds). Different letters (A–F) indicate significant differences between treatment means at *p* < 0.01.

Microgreens are 10 to 20-day-old seedlings with rich nutrient content and great potential for controlled environment agriculture. Hence, we further studied the effect of different light intensity and Se dosage on the arugula microgreen growth, yield, and Se uptake under controlled growth chamber conditions. *E. sativa* plants grew faster and taller than *D. tenuifolia*. The harvest day when the first true leaf emerged for *E sativa* and *D. tenuifolia* was 14th and 15th day, respectively. Figures 3, 4 show the growth status of plants on harvest day. Both arugulas showed a similar response to light intensity and Se dosage. HI light with the high Se doses (5–10) produced longer hypocotyls and larger and more fully expanded cotyledons than those exposed to LI light and low-dose Se (1 ppm). Interestingly, at the same Se dose, HI, with or without UVA, significantly promoted arugula growth and increased fresh weight both in *E. sativa* and *D. tenuifolia* (Figure 5A). For example, at 10 ppm Se level, the fresh weight of *D. tenuifolia* and *E. sativa* under HI increased by 16 and 13% over LI-treated microgreens. Additionally, the fresh weight of the two arugulas was not impacted by adding UVA under HI. While HI-UVA tended to produce lower values of fresh weight than HI, those were not significant. For example, at 10 ppm selenium level, the fresh weight of *E. sativa* under HI and HI-UVA was 2.63 and 2.31 g, respectively.

Moreover, at the same light intensity, the seedlings treated with 10 ppm Se had highest yield as compared to that of the 0, 1, and 5 ppm treatments in both arugula species. The *D. tenuifolia* plants subjected to the HI treatment had near 60% increased fresh weight (Figure 5B). Similarly, under the HI, the fresh weight of *E. sativa* at 10 ppm Se increased by 32% with respect to the control. Thus, the results suggested that the Se treatments had a stronger growth promoting effect than light. To confirm this, the ANOVA analysis was further performed to model the impact of light intensity and Se dosage on microgreens fresh weight. As shown in Table 1, the sum of squares (SS) value of these Se dosages was significantly larger than that of light intensity in both arugula species, even though light intensity had significant effect on microgreens yields. No clear interaction between light and Se dosage was observed.

**Table 1:** The ANOVA analysis between the effects of selenium dosage and light intensity on arugula microgreens yield.

**Species**	***Eruca sativa***		***Diplotaxis tenuifolia***	
**Source of variation**	**Sum of squares**	***P*-value**	**Sum of squares**	***P*-value**
Selenium dosage	2.459439	5.82772E-14	4.908603	3.07123E-18
Light intensity	0.59817	3.2358E-08	0.797175	3.88452E-10
Interaction	0.016805	0.896506016	0.094043	0.05857222
Within	0.18641		0.156787	

### Effects of Light Intensity and Se Dosage on Se Accumulation in Arugula Microgreens

**Figure 6:**
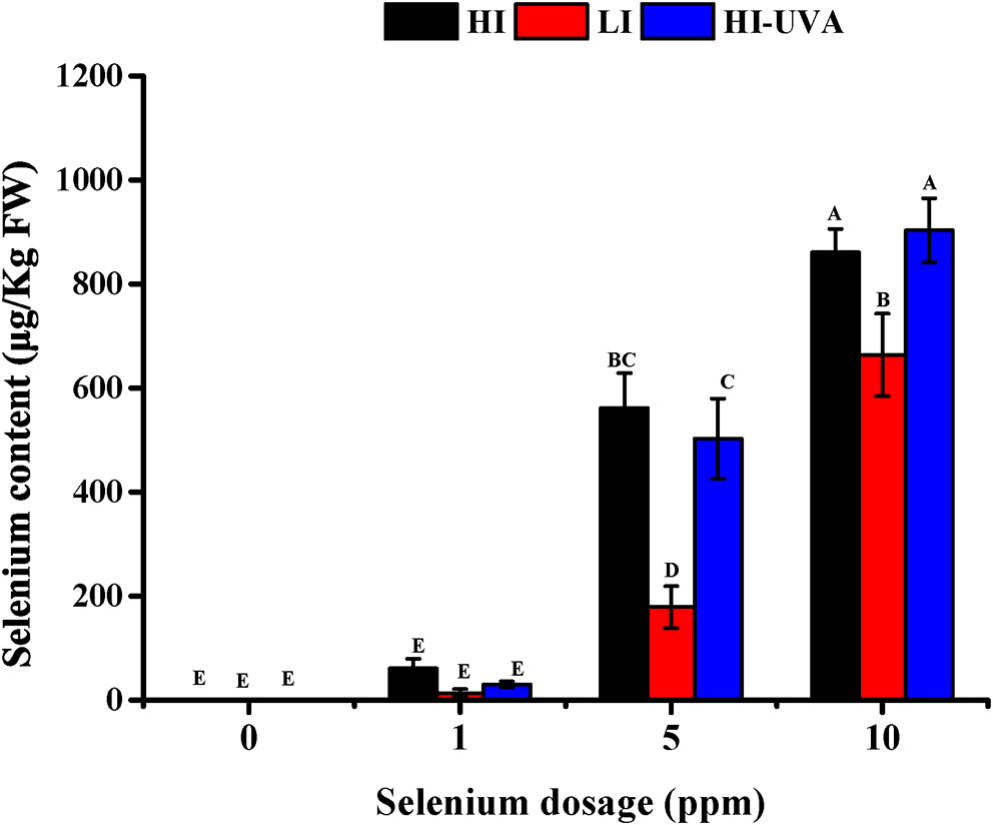
Effects of light and selenium application on the selenium accumulation in microgreens of *Diplotaxis tenuifolia* cv. Sylvetta. Plants were grown for 15 days in a growth chamber at 25°C with different light settings. HI, high-light intensity, 160 μ mol m^−2^ s^−1^ for 12 h/12 h light/dark; LI, low-light intensity, 70 μ mol m^−2^ s^−1^ for 12 h/12 h light/dark; HI-UVA, high-light intensity plus UVA (385 nm), 160 μ mol m^−2^ s^−1^ for 12 h, 40 μ mol m^−2^ s^−1^ for 2 and 10 h dark. Data presented are the means of three replications. Different letters (A–E) indicate significant differences between treatment means at *p* < 0.01.

We further examined the Se accumulation in *D. tenuifolia* microgreens. As it was expected, Se accumulation in plants was dependent on Se dosage in the hydroponic growing medium (Figure 6). For example, under HI, Se content in microgreens was 61, 634, and 844 μg/kg, respectively, when adding 1, 5, and 10 ppm Se in the growth medium. At the same Se dose, either HI or HI-UVA, enhanced Se content in microgreens as compared to LI. The correlation analysis performed between light treatment and Se accumulation in plants revealed that HI-UVA had the highest correlation index and *R*^2^, whereas the LI treatment exhibited the lowest values (Table 2). Specifically, the correlation index and *R*^2^ between HI and Se accumulation were 0.93 and 0.86, respectively, and these values between LI and Se accumulation were 0.89 and 0.79, respectively. HI had values similar to those of HI-UVA, suggesting that adding UVA has no clear effect on selenium uptake. The results evidenced a positive relationship between light intensity and Se accumulation level in *D. tenuifolia* microgreens, suggesting that light enhances the uptake of selenium in seedlings. Altogether, our results indicate that both light intensity and Se application significantly increase arugula microgreens yield and Se content.

**Table 2:** Correlation analysis between light intensity and selenium content in microgreens of *Diplotaxis tenuifolia* cv. Sylvetta.

	**HI**	**LI**	**HI-UVA**
Correlation index	0.927096934	0.894056389	0.980342943
*R* ^2^	0.859508726	0.799336826	0.961072286
Adjusted *R*^2^	0.789263089	0.699005239	0.941608428

*HI, high-light intensity, 160 μ mol m^−2^ s^−1^ for 12 h/12 h light/dark; LI, low-light intensity, 70 μ mol m^−2^ s^−1^ for 12 h/12 h light/dark; HI-UVA, high-light intensity plus UVA (385 nm), 160 μ mol m^−2^ s^−1^ for 12 h, 40 μ mol m^−2^ s^−1^ for 2 and 10 h dark*.

## Discussion

The results of the survey of the lettuce plants across various locations in the USA and Canada where commercial crops are produced showed ample Se variability. This wide range of Se values in salad greens has also been reported in other areas. For example, in Australia, Se content in lettuce also fluctuated in a wide range from 3.0 to 22.8 μg/kg (37).

This variability in Se was initially thought to be exclusively linked to the Se content in soil, as generally most reports identify soil as the key limiting factor determining the final Se content in a plant (3, 38, 39). However, the higher content in the lettuce grown in the Colorado river basin could not be explained by Se content in the soil alone, as higher Se content in soil has been reported in many of the other areas included in this study (40). Moreover, the potential higher desiccation of soils under the high annual temperatures in the Colorado river basin region should have produced negative impacts, rather than positive, on the mobility of Se and its eventual uptake by plants (41, 42). In summary, associating Se content in soil with Se in lettuce is complicated, as bioavailability of Se in soils is governed by physicochemical factors including adsorbing surfaces, pH, chemical composition, and redox status (38, 39, 42–44).

Thus, early in this study, we learned that the role of other factors, in addition to soil content, has more impact than initially expected. In this study, the lettuce plants collected were grown in different environmental conditions and were subjected to different practices such as the irrigation modality. In this regard, leafy greens accumulated more Se when they were subjected to overhead sprinkle irrigation than when subjected to flood irrigation (45). The explanation for this may be attributed to sprinkle irrigation water prompting a change in the dynamic photosynthesis and respiration of plants or by reducing heat stress. In the latter cases, light intensity appears to have a key role. Intensity within certain spectral ranges was also found to drive stomatal conductance of lettuce (46). Ordinarily, selenate, SeMet, and selenomethionine-Se-oxide (SeOMet) are moved in the xylem (47, 48), which also reveal the relevance of light during the growing cycle and the effect of transpiration of plants on the accumulation of Se in plants. Therefore, light intensity was the factor that we hypothesized could explain the large variability of Se content and/or the induction of larger accumulation of Se in plants. Interestingly, the Sonoran Desert area, which covers the Colorado river basin region, consistently shows the highest annual accumulation of incident solar radiation in North America with as high as 50–70% more than that of other regions covered in this study such as the mid-Atlantic and the Northeast Coast (36).

The variability of Se content has relevance from a higher level nutritional perspective. Considering the importance of the RDA of Se for human health, the observed variable Se content underscores the need for more accurate food composition tables. Food composition databases rarely account for environmental factors such as soil chemistry and light intensity. Accounting for these factors and their influence on variability, have been identified as a major need to improve nutrient requirement guidelines and nutritional epidemiology (49). The United Nations Food and Agriculture Organization (FAO) has advocated for more available food composition data that would precisely account for differences in varieties/cultivars and their adaptations to meteorological conditions (50). The results of this study support this view. However, an optimal RDA for Se and more accurate determination of food composition databases are further obfuscated by several factors. A given food may have Se in different quantity and forms of Se complexes with different bioavailability such as selenoglucosinolates (51) depending on environmental influences specific to where the plant is grown. In addition, the specific needs of an individual for Se may vary as a function of their specific lifestyle or family history (21).

Aside from the large data variability, this study suggested a trend toward low levels of Se in lettuce grown in North America, similar to reports by others in countries considered to have low Se levels in soils. In Japan, lettuce has been reported to have Se levels as high as 7 μg/kg (52), while in New Zealand, an average of 2.7 μg/kg has been reported (53). These are low levels compared to those reported in areas of Europe such as Croatia (27) and Slovakia (54) where lettuce Se content was found to be in the range of 9–14.5 μg/kg. Despite the low levels, the high consumption per capita [nearly 26 pounds per capita; (34)] may still make lettuce a relatively good contributor of Se in the diet. Introducing Se in the lettuce production system through fertilization may be a feasible option, taking into consideration that lettuce has been reported not to accumulate Se to toxic levels. In fact, when lettuce was subjected to levels close to human toxicity, the plants did not survive (55).

On the contrary, some forage *Brassica* species can accumulate Se to concentrations that may be toxic to animals (45, 56). The increasing interest in consumption of Brassicaceae plants due to the high content of antioxidant compounds and in particular of sulfur-based compounds such as glucosinolates makes this family of plants of great economic/nutritional value in society of today. The sulfur content of different Brassicaceae tissues increases consistently, as the Se content of plants increased in a synergistic interaction between selenate and sulfate (57). In a recent study comparing lettuce and arugula tolerance to Se, the accumulation of Se was 4- to 6- fold higher in arugula (*E. sativa*) and the authors attributed this difference to a more efficient antioxidant defense system of arugula (58).

The significant decline of Se in arugula, following a 90% sunlight blockage for just a short period of time (1 week), revealed how critical light intensity can be before harvest of leafy greens to obtain Se-enriched leafy greens. However, more study is needed to identify physiological explanations for this remarkable change and as to determine whether the plant accelerates Se uptake solely to cope with high photo-oxidation and volatilization conditions or whether changes in relative humidity had any impact. The results also showed the large difference between the *D. tenuifolia* and *E. sativa*, which have also been reported in a study that enriched irrigation water to biofortify leaves with Se (59). The latter report showed that the plants reached a plateau level of Se, but the researchers did not verify whether light had any impact on this plateau.

To the best of our knowledge, this study confirms this effect of light for the first time under controlled environmental conditions. Our results showed the significant effect of light alone in the presence of Se at 1–10 ppm. This result could be explained by earlier findings that showed Se alters the dark respiration (and not photosynthesis) of *E. sativa* (60). Furthermore, and despite the broad assumption that Se is not essential in plant physiology, the experience in Finland with the sodium selenate supplementation of fertilizers has shown an association of Se content with the vigor and healthy growth of plants (61). The results in this study revealed a marked effect of 1–10 ppm Se application to increase yields and suggests that Se in certain dosage range promotes plant growth. In future studies, we aim to investigate whether the Se-positive effect on growth observed in this study is species or seed composition dependent. To complement our findings, further study is also warranted to determine implications of Se content on postharvest quality and shelf-life of salad vegetables, given the potential role of Se to enhance antioxidant activity and inhibit the biosynthesis of ethylene (62). The combination of Se and light under controlled conditions may also cause some additional changes in bioactive composition (sulfur-bound compounds). This could, in turn, result in changes in sensory perception, which also require further investigation.

## Conclusion

Our results demonstrated that light intensity has significant positive effects on the accumulation of Se in salad greens, both in Se non-accumulating (lettuce) and Se accumulating (arugula) plants. In controlled growing conditions, this effect was found to be unaffected by the concentration of Se in the substrate above a certain minimum level (1 ppm), as no interaction between light effects and Se treatment effects was identified. Interestingly, we also found that regardless of the light intensity, the Se content in the substrate increases the yields of arugula. Under controlled condition, adding different doses of Se in the growth medium can dramatically increase the Se content in salad greens and produce functional salad greens with desired Se contents for different populations. This study provides the controlled environment industry with key information to produce salad greens with targeted Se contents. It also contributes with evidence to justify the revision of food composition databases to account for large Se variability.

## Data Availability Statement

The raw data supporting the conclusions of this article will be made available by the authors, without undue reservation.

## Author Contributions

XZ contributed to the investigation, methodology, and writing. CS contributed to the resources, writing—review, and editing. JH contributed to the validation, writing—review, and editing. TY and JF contributed to the conceptualization, writing—review and editing, supervision, and funding acquisition. All authors have read and agreed to the published version of the manuscript.

## Funding

This study was supported by the office of the Vice-President of Research, The University of Arizona, and the United States Department of Agriculture-Agricultural Research Service (USDA-ARS Grant No. 8042-43000-016-00D) National Program 306.

## Conflict of Interest

The authors declare that the research was conducted in the absence of any commercial or financial relationships that could be construed as a potential conflict of interest.

## Publisher's Note

All claims expressed in this article are solely those of the authors and do not necessarily represent those of their affiliated organizations, or those of the publisher, the editors and the reviewers. Any product that may be evaluated in this article, or claim that may be made by its manufacturer, is not guaranteed or endorsed by the publisher.
