# Supplementary material for: Nutrition by Design: Boosting Selenium Content and Fresh Matter Yields of Salad Greens With Preharvest Light Intensity and Selenium Applications
Source: Front Nutr. 2022 Jan 5;8:787085. doi: 10.3389/fnut.2021.787085 (PMC8766809; doi:10.3389/fnut.2021.787085)
Supplement: Supplementary file 1 [file Data_Sheet_1.PDF]

Supplementary Material

1 Supplementary Figures and Tables

1.1 Supplementary Figures

Supplementary Figure 1

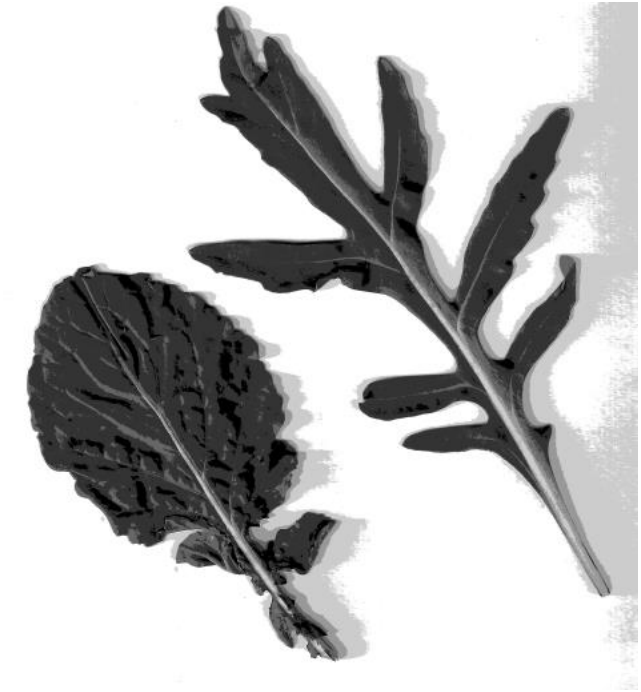

FIGURE S1: Leaf morphology of *Eruca sativa* (Salad rocket) cv. 'Astro' (left side) and *Diplotaxis tenuifolia* (Wild rocket) cv. 'Sylvetta' (right side). Source: J.M. Hamilton

Supplementary Figure 2

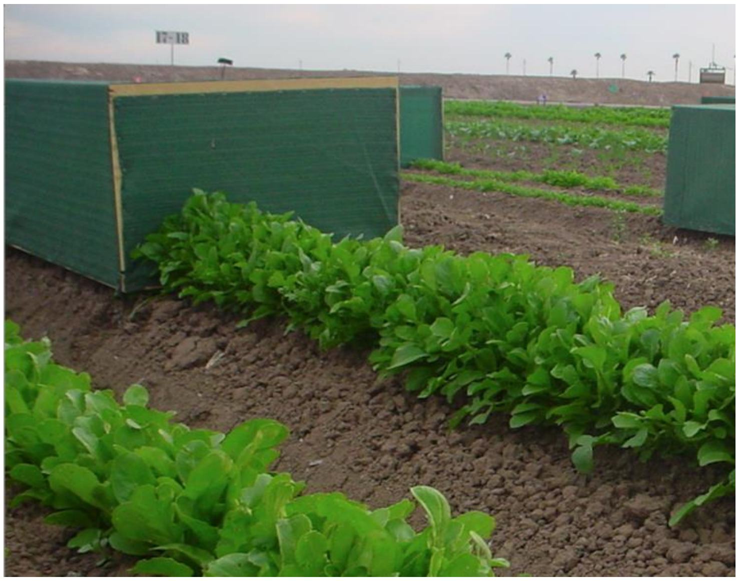

FIGURE S2. Box with nylon cloth used to reduce 90% sunlight reaching *Eruca sativa* plants during the last 7 days before harvest.

1.2 Supplementary Tables

Table S1: Source location, number of units and type of lettuce sampled to determine the content of selenium in lettuce grown in the USA. Lettuce from Yuma, AZ, Imperial valley, Cochella valley and Bard, California are irrigated with water from the Colorado river.

Location	Number of samples	Type of lettuce
Yuma, AZ *	28	IC, RN, BO, GL, RL
Bard, CA *	32	IC, RN, BO, GL, RL
Imperial valley, CA *	20	IC, RN, BO, GL, RL
Cochella valley, CA *	24	IC, RN, BO, GL, RL
Huron valley, CA	4	IC, RN, GL, RL
Las Cruces, NM	2	IC, RN
Salinas, CA	4	IC, RN, BO, GL
East Lansing, Michigan	4	RN, BO, GL, RL
Columbus, Ohio	3	BO, GL, RL
Ithaca, NY	6	IC, RN, BO, GL, RL
Montreal, Canada	4	IC, RN, BO, RL
Alamosa, Colorado	4	IC, RN, GL, RL
Albuquerque, NM	2	GL, RL
Santa Maria, CA	2	IC
Cumberland County, NJ	5	IC, RN, BO, GL, RL

* Indicates lettuce crops irrigated with Colorado river water, IC=iceberg lettuce, RN= romaine lettuce, BO=boston lettuce, GL= green leaf lettuce, and RL= red leaf lettuce.

Table S2: Selenium concentration (ppm) in soil of selected nearby locations were lettuce samples were sourced from.

Place (County)	Mean	Std. Dev	Min	Max
Yuma, AZ *	0,129	0,0035	0,100	0,355
Imperial, CA *	0,238	0,159	0,100	1,295
La Paz, AZ *	0,168	0,054	0,100	0,657
Riverside, CA	0,173	0,147	0,100	1,376
Monterrey, CA	0,427	0,257	0,101	2,224
Dona Ana, NM	0,140	0,057	0,100	0,497
Fresno, CA	0,298	0,318	0,0051	3,341
Tompkins, NY	0,224	0,048	0,104	0,338
Cumberland, NJ	0,625	0,270	0,292	2,101

Source: United States Geological Survey, 2021().

* Indicates information of soil in areas irrigated with Colorado river water

Table S3. Period daily-averages for temperature (ºC) relative humidity (RH) and solar radiation (SR) during field cultivation of arugula species in Yuma, AZ, USA.

	Early crop		No cultivation			Late crop	
	**Oct**	**Nov**	**Dec**	**Jan**	**Feb**	**Mar**	**Apr**
**SR Langley day $^{-1}$**	440	329	253	291	388	518	649
**Mean (ºC)**	22.2	18.1	12.5	11.6	14.0	17.6	21.5
**Mean RH %**	36	46	55	50	39	33	29

Source: AZMET. The Arizona Meteorological Network, Yuma Data Reports (https://cals.arizona.edu/azmet/02.htm)

Table S4. Shade cloth material specifications, used during field cultivation of arugula species in Yuma, AZ. USA

**Expected light reduction**	60%	90%
**Measured light reduction**	64%	88%
**$g - m^{-2}$**	20.4	25.84
**Material thickness**	0.75 mm	1.15 mm
**Temperature reduction**	˜1ºC	˜2ºC
**Color (Pantone solid)**	5487M, 555M, 5555M	5625 M, 5555M, 5555 M
